# A Brief Review of Multipath TCP for Vehicular Networks

**DOI:** 10.3390/s21082793

**Published:** 2021-04-15

**Authors:** Luomeng Chao, Celimuge Wu, Tsutomu Yoshinaga, Wugedele Bao, Yusheng Ji

**Affiliations:** 1 Graduate School of Informatics and Engineering, The University of Electro-Communications, Tokyo 1828585, Japan; chaolm@comp.is.uec.ac.jp (L.C.); yoshinaga@uec.ac.jp (T.Y.); 2 School of Computer Science and Information Engineering, Hohhot Minzu College, Hohhot 010051, China; wugedele2018@163.com; 3 Information Systems Architecture Research Division, National Institute of Informatics, Tokyo 1018430, Japan; kei@nii.ac.jp

**Keywords:** MPTCP, multipath routing, vehicular networks

## Abstract

Multipath TCP (MPTCP) is one of the most important extensions to TCP that enables the use of multiple paths in data transmissions for a TCP connection. In MPTCP, the end hosts transmit data across a number of TCP subflows simultaneously on one connection. MPTCP can sufficiently utilize the bandwidth resources to improve the transmission efficiency while providing TCP fairness to other TCP connections. Meanwhile, it also offers resilience due to multipath data transfers. MPTCP attracts tremendous attention from the academic and industry field due to the explosive data growth in recent times and limited network bandwidth for each single available communication interface. The vehicular Internet-of-Things systems, such as cooperative autonomous driving, require reliable high speed data transmission and robustness. MPTCP could be a promising approach to solve these challenges. In this paper, we first conduct a brief survey of existing MPTCP studies and give a brief overview to multipath routing. Then we discuss the significance technical challenges in applying MPTCP for vehicular networks and point out future research directions.

## 1. Introduction

In the past, people have mostly relied on newspapers, magazines, or books to acquire the information they need. After that, with the development of electromagnetic technologies, people began to utilize some extra options of electric tools such as television and radio to acquire information. The communication world is constantly and quickly changing due to the evolvement of information and communication technology and it gradually changes and affects our life. Nowadays, we can chat with people from most places in the world via a smartphone. There are many transmission protocols, such as user datagram protocol (UDP) [1], transmission control protocol (TCP) [2], and stream control transmission protocol (SCTP) [3], to support communications. TCP and SCTP can provide reliable transmissions. However, their transmission rates are very limited. Therefore, we need a solution to solve this challenge.

In the current era, many smart phones and mobile devices are equipped with dual interfaces, even with multiple interfaces. Cellular network technology and Wi-Fi are pervasively utilized. In January 2013, to overcome the limitation of conventional single path transmission rate, the Internet Engineering Task Force (IETF) published the multipath TCP (MPTCP) specification for TCP extensions [4]. Multipath TCP allows an end host to transmit data across a number of TCP subflows simultaneously on one TCP connection. MTCP is designed by considering the following points:

- Improving throughput: MPTCP’s throughput is larger than a single path TCP by sending the data through multiple paths simultaneously;
- TCP friendliness: MPTCP does not incur a negative effect on the conventional single path TCP;
- Balancing congestion and improving resiliency: MPTCP utilizes the least congested path, and therefore is able to shift the flows from congested or failure path to the least congested path.

These advantages have attracted attention from academia and the industry. Many studies in regards to MPTCP were conducted under various topics, including data center communications, smart phone applications, energy efficiency, vehicular networks, and so on. On September 2013, Apple released iOS7 which included the commercial deployment of MPTCP [5]. At IETF’93 in Prague, Seo first presented that Korean Telecom would provide a service that enables smartphone users to reach a bandwidth of up to 1Gbps on smartphones [6]. They provide the service by combining fast long term evolution (LTE) and fast Wi-Fi. The vehicular Internet-of-Things (IoT) systems are equipped with many sensors, including a camera and radar, and hence generate a large amount of data. Meanwhile, emerging cooperative autonomous cars and intelligent transport systems demand unprecedented high transmission speed and a high degree of robustness. Most of the current vehicular IoT systems utilize single path to transmit data between vehicles, which hardly reaches the demand of data transmission speed of autonomous driving. Moreover, in the context of mobility, the IP address changes when users move out of an access point’s coverage range or is disconnected when using single path TCP. The unfinished data will be stopped or the whole transmission will be reset. Meanwhile, vehicular networks have a feature of complex and dynamic topology. Therefore, in order to achieve a more efficient and robust vehicular IoT system, MPTCP is considered to be a promising approach to reach this goal since it can transmit data across multiple paths to improve transmission throughput. In addition, it is able to shift the flows to other paths when the transmitting path is interrupted. Furthermore, it increases resilience to respond to constant topology changes. On the other hand, because vehicular networks conduct wireless communication, it may cause communication interferences. Therefore, it is pointed out that selecting the proper transmission paths is also very important [7]. Studies regarding multipath routing [8,9] were widely conducted to provide efficient and robust transmission paths. Furthermore, there are already some works to combine multipath routing with MPTCP to improve the performance in vehicular networks [10,11]. However, implementing MPTCP in vehicular IoT systems still face several key challenges. Firstly, utilizing different communication methods such as LTE or IEEE 802.11p would incur different transmission latencies. This could result in bufferbloat at receivers, thus impacting the application level performance. Secondly, most vehicular applications require high quality-of-service (QoS) demands, so the MPTCP architecture need to be further improved to reach the goal. Third, due to the feature of constant movement of vehicles, it is hard to maintain a stable network topology in vehicular networks. Therefore, it is important to design an efficient routing protocol to offer stable communications.

Due to the aforementioned challenges, a new MPTCP architecture that could improve communication efficiency with low overhead is of great interest. In this paper, we conduct a survey on the technical challenges and existing solutions regarding MPTCP, and the implementation of MPTCP in vehicular networks. We also give a brief introduction in regards to multipath routing in vehicular networks, which is the basis of achieving multipath data transmissions in highly mobile environments. The main contributions of this paper are as follows:

- To the best of our knowledge, this is the first work that presents recent advances of MPTCP in vehicular networks and multipath routing in vehicular networks;
- We discuss both the technical issues of applying MPTCP in vehicular networks, and necessary improvements to support MPTCP applications in vehicular networks;
- We point out the future research directions related to applying MPTCP in vehicular networks.

The remainder of this paper is organized as follows. We first introduce fundamentals of TCP and MPTCP in Section 2. We review the two important components of MPTCP, namely, the scheduler and congestion control (CC) in Section 3. In Section 4 we introduce recent studies that combine MPTCP with other techniques and some research efforts in applying MPTCP for IoT systems. We present the studies that employ MPTCP in vehicular networks in Section 5. The research efforts on multipath routing in vehicular networks are presented in Section 6. We discuss future research directions in Section 7, and conclude this paper in Section 8.

## 2. TCP and MPTCP

### 2.1. Overview of Studies on TCP

Nowadays, most of applications utilize TCP to transmit data since TCP has a retransmission mechanism to retransmit lost packets, which ensures that all the data packets could be correctly delivered to the receiver. TCP also utilizes a congestion control algorithm (CC) [12] to adjust sending rate based on packet loss or delay in order to avoid network congestion. In contrast, UDP does not conduct retransmissions and congestion control. Therefore, most of the voice over IP (VoIP) applications utilize UDP to provide real-time and uninterrupted call. Errors such as packets losses have minor impacts on the audio output and are usually not noticed. Quick UDP Internet Connection Protocol (QUIC) [13,14] is developed by Google. The QUIC specification draft has been released by IETF on January 2021. QUIC runs on top of UDP. It combines the functions of HTTP/2, TLS and TCP. For example, QUIC handles the flow control, crypto, and HTTP. The design goal of QUIC is to reduce the connection establishment time by eliminating a lot of round trips and realize reliable transmissions without sacrificing security. Conventional TCP and UDP live in kernel space so that it is very hard to make fundamental changes to the protocol. In contrast, QUIC is implemented in user space, and therefore it can be customized to specific network scenarios more easily. Similar to HTTP/2, QUIC supports multi-streaming. Moreover, QUIC can solve a head-of-line blocking problem which HTTP/2 cannot handle. However, QUIC has not been massively deployed and further investigations are required to verify its effectiveness and maturity. One of the most important concerns about QUIC is security.

It is pointed out that standard TCP underutilizes the bandwidth greatly due to its additive increase/multiplicative decrease (AIMD) scheme [15]. There have been many studies related to TCP to improve its bandwidth utilization. They are mainly classified into three types, respectively, loss-based, delay-based, and hybrid approach. Loss-based approaches such as TCP CUBIC [16], STCP (scalable TCP) [17], and BIC-TCP (binary increase congestion control) [18], adjust the sending rate based on the packet loss. Among them, TCP CUBIC congestion control is currently the default congestion control mechanism in many Linux systems. When a packet loss occurs, CUBIC increases the cwnd value drastically when the TCP cwnd size is far from reaching the maximum value, and increases cwnd slowly when it reaches near the maximum value. In another word, there is a larger increase at a lower cwnd value, and smaller increase at a higher cwnd value. This results in different effects on different congestion conditions. The delay-based approach such as FAST TCP [19] and bottleneck bandwidth and round-trip propagation time (BBR) [20] adjusts the sending rate based on round trip time (RTT) variations. The BBR congestion control algorithm is developed by Google. It utilizes the estimated real-time bandwidth and round trip time to determine the data transfer rate, which would result in a much more accurate prediction of the congestion. A model is built to determine the transfer rate to maximize the data rate, which always adjusts the model based on the real time congestion measurement. BBR considers that the loss-based congestion control is ineffective, since packet losses can occur due to many factors, in addition to congestion. When the estimated round trip time starts to increase, BBR knows the congestion exists. However, BBR results in a high packet retransmission rate in shallow buffer and lossy networks. The hybrid approach [21] considers both the loss and delay in the control process of congestion window.

It was argued that a blameless TCP protocol has to meet the following three requirements, specifically, efficiency, RTT fairness, and TCP fairness. However, it is difficult to satisfy all these three requirements. While STCP and BIC-TCP can achieve a relatively higher sending rate, they cause RTT unfairness and TCP unfairness. Delay-based FAST TCP can satisfy the first two requirements, but it still causes TCP unfairness. In order to satisfy all the requirements, Tan et al. [21] proposed an approach called CTCP (compound TCP). The CTCP adds a delay-based component to the standard TCP, and combines the delay signal and loss signal to adjust the congestion window. The consideration of RTT in the congestion control makes CTCP better handle the TCP unfairness in a lossless communication environment.

The above-mentioned approaches generally adopt a pre-determined policy with fixed system parameters for implementing congestion control. They are incapable of satisfying the performance requirements under dynamic network environments. To make the congestion control protocol adapt to dynamic environments, Li et al. [22] integrate a reinforcement-based Q-learning framework with TCP, called QTCP. In QTCP, the congestion control gradually converges to the best rule without prior knowledge of the new environment. Hence, QTCP is capable of adjusting congestion control corresponding to a new network scenario. Moreover, they developed a generalization-based Kanerva coding function approximation approach to reduce the computation complexity and necessary state space, therefore reducing the training time.

In most of the TCP variants, the congestion window starts from a small initial window (IW), specifically, 2, 4, or 10. For a short flow, TCP suffers from the slow start problem as the flow is still in additive increase state before the session ends. For a long flow service, the conventional manually- and statically-configured congestion control is not able to fulfill the latest network performance requirements. To address the above two challenges, Nie et al. [23] proposed a TCP reinforcement learning (RL) technique, called TCP-RL. The TCP-RL dynamically adjusts the IW and congestion control to improve TCP flow transmission efficiency. Such learning based TCP variants are able to provide a higher throughput than rule-based TCP variants. The above-mentioned research efforts are summarized in Table 1.

### 2.2. Overview of Multipath TCP

**Figure 1:**
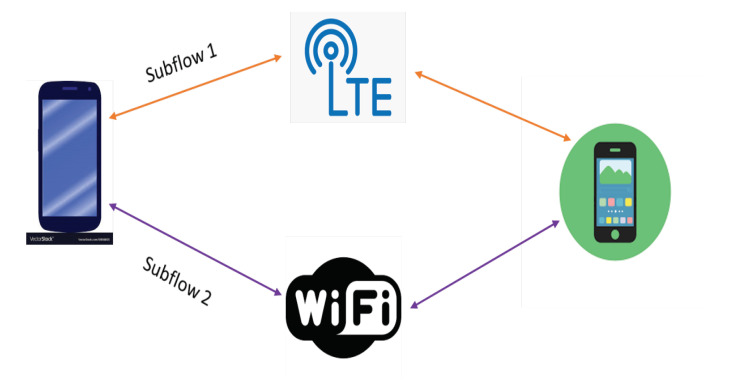
Multipath TCP.

Nowadays, most user devices have multiple interfaces and server machines are multihomed. Meanwhile, in order to ensure reliability, data center networks utilize extra infrastructures to provide many redundant paths between any two servers. However, conventional single path TCP cannot utilize these multiple paths efficiently due to failing to couple data streams efficiently. To overcome this limitation, multipath TCP (MPTCP) is developed. MPTCP allows the end-hosts to transfer a single data stream through multiple paths. It standardizes and implements a deployable multipath extension to TCP. As shown in Figure 1, the end hosts can transmit data across a number of TCP subflows simultaneously in a MPTCP connection. Before MPTCP, there were many attempts that attempted to transmit data across multiple path simultaneously. For example, the SCTP [3] is a transport layer connection-oriented protocol, which can achieve reliable transmissions. Similar with TCP’s three-way handshake, SCTP conducts a four-way handshake to establish the association between two end points. TCP is vulnerable to denial of service (DoS) attacks. However, SCTP can increase security by utilizing the cookie mechanism and is able to better respond to this problem. SCTP supports multi-homing, so that each of its end points can hold multiple IP addresses. Therefore, multiple paths can exist in one SCTP association. However, only the primary path is allowed to transmit data while other paths are for backup use. SCTP combines the advantages of TCP and UDP. Concurrent multipath transfer using SCTP (CMT-SCTP) [24] is an extension of SCTP, which supports multi-path data transmission within a SCTP association. Packets are allocated to different path by round robin (RR) manner in CMT-SCTP. However, this method will bring packet disordering when encountering heterogeneous paths. MPTCP architectural guideline was discussed in [25]. In [26], Paasch et al. implemented experiments and showed that MPTCP is able to switch the transmission path between different paths with only a small delay.

In January 2013, the Internet Engineering Task Force (IETF) published the MPTCP specification for TCP extensions [4]. MPTCP is mainly designed to achieve three purposes, specifically, improving throughput, being TCP friendly, balancing congestion, and improving resiliency in the event of failure. In both the academic and industrial world, many studies regarding MPTCP were conducted.

**Figure 2:**
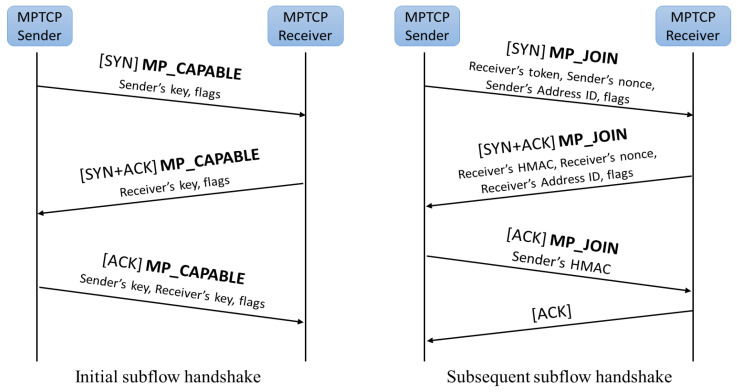
Multipath TCP initial subflow handshake and subsequent subflow handshake.

In MPTCP, each subflow is a standard TCP connection. As illustrated in Figure 2, in the MPTCP connection establishment state, an initial subflow has to be established. Similar with TCP, it also conducts a three-way handshake to initiate an initial subflow. At first, the sender sends the SYN packet that includes the MP_CAPABLE option, sender’s key, and flags to the receiver. The MP_CAPABLE option indicates that the sender supports MPTCP and wants to start MPTCP. Then, if the receiver also supports MPTCP, it will reply with a SYN/ACK packet that also includes the MP_CAPABLE option, receiver’s key, and flags. Then, the sender replies with a final ACK that includes the MP_CAPABLE option, sender’s key, receiver’s key and flags, and successfully sets up a MPTCP capable session. However, at the SYN/ACK sending phase, if the receiver does not support MPTCP or does not add a MP_CAPABLE option to a SYN/ACK packet, the MPTCP session returns to a regular TCP. The exchange of keys in this initial subflow establishment stage also provides cryptographic information for subsequent subflow establishment. The cryptographic information is used by the subsequent subflow to identify which MPTCP connection it wishes to join. After the initial subflow is established, as illustrated in Figure 2, a new subflow can be added to the existing MPTCP connection by conducting a four-way handshake. Firstly, MPTCP initiates the subsequent subflows by sending the MP_JOIN option. Besides the MP_JOIN option, the SYN packet also includes the cryptographic information (receiver token), sender’s random numbers (nonce), sender’s address ID, and flags. Then the receiver replies with the SYN/ACK packet that includes the receiver’s hash-based message authentication code (HMAC), receiver’s nonce, receiver’s address ID, and flags after receiving the SYN packet. After that, the sender replies with ACK that includes the MP_JOIN option and sender’s HMAC. At last, the receiver sends ACK to successfully establish subflow. The HMAC utilizes the keys exchanged in the MP_CAPABLE and nonce (random numbers). MPTCP has a connection-level sequence number and subflow-level sequence number. Connection level sequence number is the data sequence number of packets that is distributed to each subflow before. The subflow-level sequence number is the sequence number of packets on each subflow, which is similar with the sequence number of conventional TCP. The subflow-level sequence number is mapped to the connection-level sequence number, which will enable packets to be transmitted through different paths.

As mentioned earlier, in the standard MPTCP connection establishment phase, an initial subflow is needed for exchange signaling information between the sender and receiver in order to enable the data transmission through multiple paths. Considering the connection could be affected by the default path which is suffered from packet losses, Amend et al. [27] proposed robust connection establishment (RobE), a solution that uses additional potential subflows to improve the robustness of establishing multipath data transmissions.

After the establishment of initial subflow, the speed of starting up the other subflows is considered to be an important factor for MPTCP performance. In [28], Chen et al. conducted an experiment with two-flow MPTCP. They showed that the delayed startup of additional flows results in low bandwidth utilization for small data. For large data, the concurrent exploitation of Wi-Fi and cellular would result in performance degradation due to bufferbloat. Furthermore, they studied the influence of bufferbloat to MPTCP performance, and provided a solution to mitigate the influence of severe bufferbloat. Nikravesh et al. [29] first put great efforts on the MPTCP performance and cross-layer interactions by conducting a real-word experiment. They discussed the multipath-aware content delivery network (CDN) server selection problem. For example, for small size file transfers, selecting the servers with minimum latency is reasonable. In contrast, for large size file transfers, selecting the servers that maximize the overall bandwidth is more important. Meanwhile, based on the user trial, a new flexible system for mobile multipath called MPFlex was designed and implemented in this work. The MPFlex employs multiplexing technology and decouples the scheduling algorithm and OS protocol implementation. Multiplexed connections (MCs) are pre-established by only one handshake, instead of conducting handshakes for every subflow establishment, and stayed at a connected state. Thus, the startup time can be reduced largely. Meanwhile, as compared with MPTCP, the scheduler implementation of MPFlex had been greatly simplified by decoupling it from OS, avoiding the entire kernel upgrade for each software update of MPFlex. MPFlex also used a policy engine to provide a higher level control on how to utilize multiple paths according to user requirements. Chaturvedi et al. in [30] proposed a security protocol for multipath TCP, namely secure connection multipath TCP (SCMTCP). The SCMTCP has three modules, including SCMTCP with MP_CAPABLE (SCMTCP_MPC), SCMTCP with MP_JOIN (SCMTCP_MPJ), and SCMTCP with ADD_ADDR (SCMTCP_ADA). SCMTCP_MPC generates a private key and public key. At the initial subflow establishment stage, the public keys will be exchanged along with the MP_CAPABLE option. SCMTCP also employs a third-party certificate authority to ensure the successful exchange of public keys. SCMTCP_MPC is for protecting MPTCP from man-in-the-middle attack. When adding a new subflow to the existing MPTCP connection, SCMTCP_MPJ also generates a unique key and exchanges key information along with MP_JOIN. SCMTCP_MPJ is for protecting MPTCP from denial of service. Likewise, SCMTCP_ADA also generates a unique key and exchanges it along with ADD_ADDR.

As mentioned above, improving the throughput is one of the main goals in MPTCP. Fakhimi et al. [31] proposed a Q-learning-based MPTCP algorithm. The main idea is to find out a policy to select the best combination of proper interface and congestion control method according to different network conditions so as to maximize the overall throughput. Based on the Q-learning algorithm, the sender decides whether to use single path or multiple paths for data transmissions. Congestion control mechanisms, such as opportunistic linked increases algorithm (OLIA) [32], Cubic [16], and Westwood [33] are discussed. In [34], Jin et al. proposed a path selection method based on throughput prediction and available bandwidth for MPTCP. Firstly, they studied the effects of path characteristics for MPTCP throughput. Then they analyzed the reasons why a bad path affects MPTCP performance seriously. They consider that it is not necessary to utilize MPTCP when the best single path is enough. This is because in that case, the improvement of bandwidth will be very limited by aggregating bandwidth of multiple paths due to TCP friendliness requirements. Moreover, inefficient paths may bring negative effects on overall throughput due to their longer RTTs and higher loss rates. However, this study uses a static method and further studies are required to apply the idea in dynamic environments.

MPTCP exposes standard socket API to applications. However, some applications fail to fully manage the multiple paths. To overcome this limitation, Hesmans et al. [35] proposed a MPTCP path manager that enables applications to control multiple paths of MPTCP. The path manager is separated into data plane and control plane. The data plane remains in kernel for data transmissions. The control plane is for managing the subflows. A new interprocess communication mechanism is proposed to enhance the interaction between the applications and MPTCP kernel. The mechanism behaves as a bridge to connect the in-kernel path manager with subflow controller running in userspace. This brings great convenience to control different paths by a subflow controller.

Voice recognition technology has made remarkable progress in recent years and is applied in many devices such as car navigation and smartphones. In [36], Tran et al. studied the performance of voice-activated applications in the context of MPTCP environments by utilizing the MONROE platform which is based on container technology. In order to satisfy the restriction of the MONROE platform, they port the MPTCP Linux code into the Linux kernel library. Meanwhile, since most of the popular voice recognition systems are closed source, they modified iperf3 to overcome this limitation. Results show that MPTCP plays a role in enhancing the performance of voice-activated applications. As the transmission delay is a key performance parameter for voice recognition, it could be affected more easily by network condition in single path TCP. In MPTCP, the scheduler can shift the data transmissions from a weak signal strength subflow to a stronger one.

Besides MPTCP, there are also some alternatives that can realize multiple paths transmission such as multipath QUIC (MP-QUIC) [37] and multipath transmission model for datagram congestion control protocol (MP-DCCP) [38]. MP-QUIC is the extension of QUIC, which enables multipath transmission on one QUIC connection. In [37], Quentin et al. implemented some experiments and show that MP-QUIC outperforms MPTCP both on lossy scenario and non-lossy scenarios. However, the effectiveness of MP-QUIC needs to be further verified in various scenarios. Similar with UDP, the datagram congestion control protocol (DCCP) is a transport layer protocol on the top of IP. It includes a congestion control mechanism and acknowledgement (ACK) by the receiver when receiving packets. However, the sender does not retransmit the lost packets. DCCP is very flexible. It can switch its options and feature even during transmissions. The multipath datagram congestion control protocol (MP-DCCP) is based on DCCP, and it supports the use of different congestion control algorithms. Similar with UDP, MP-DCCP does not consider retransmissions. Amend et al. proposed an IP-compatible multipath framework for heterogeneous access networks by utilizing the MP-DCCP in [38]. The framework realized IP compatibility by integrating virtual network interfaces with MP-DCCP. The MP-DCCP supports multipath transmission across multiple DCCP tunnels simultaneously. MP-DCCP measures the capacity of each tunnel based on loss rate, bandwidth and round trip time, and schedules the data that entered from virtual network interfaces across different tunnels. A scheduling module and reordering module are applied to adjust the delay difference of tunnels for MP-DCCP.

### 2.3. Comparison of Single Path TCP and MPTCP

To prove the advantage of MPTCP, many works were conducted to compare the performance of single path TCP (SPTCP) and MPTCP. At the very beginning, MPTCP is designed for improving the efficiency of data center communications. In [39], Raiciu et al. proposed an approach to replace TCP with MPTCP in datacenter networks. They investigate how each parameter of MPTCP influences the network performance and compare MPTCP performance with TCP. The influence metrics include the network topology, number of subflows, traffic matrix, and congestion control. Firstly, the results show that MPTCP can achieve a 90 percent bandwidth utilization with two subflows and eight subflows, for VL2 and FatTree network topology, respectively. Second, the experiment shows that the number of subflows also has an effect on the throughput performance. At least for the permutation traffic matrix, eight subflows are sufficient to achieve 90 percent throughput utilization. The effect of different congestion control mechanisms on network performance are also studied. They compared MPTCP’s linked congestion control mechanism with uncoupled TCP and equally weighted TCP (EWTCP). Lastly, they evaluated the performance of MPTCP in dual-homed FatTree (DHFT) scenario, a dual-homed variant of FatTree topology. The results show that MPTCP not only improves throughput, but also improves robustness with the same cost.

Chen et al. [40] conducted a web traffic measurement to compare the performance of single path TCP and MPTCP over Wi-Fi and cellular networks. Since different web traffics can have different traffic loads, they put their efforts to analyze the relationships of network factors and application level performance. They consider file downloading traffics with different file sizes, specifically 8 KB, 64 KB, 512 KB, 4 MB, 8 MB, 16 MB, and 32 MB. When the Wi-Fi is the default path, the MPTCP download performance has no obvious improvement compared with the SPTCP. For small size file download (such as 64 KB size) scenarios, the single path over Wi-Fi provides the best performance. Instead, for slightly longer traffic flows, single path over LTE becomes the best choice. The reason is that the Wi-Fi has a short RTT and high loss rate, while the cellular network has a low loss rate. The small size files can be completed very quickly by Wi-Fi. However, when the file size becomes larger, the retransmission rate will increase due to the high loss rate of Wi-Fi packets. In this case, the cellular network will result in a better performance due to its low loss rate of data packets. For 4-path MPTCP, the small size files can be quickly completed by two Wi-Fi subflows without utilizing cellular communications. Likewise, the cellular will be playing a leading role when the file size becomes larger. The effects of many factors on latencies are also evaluated. The factors include the number of subflows, congestion controllers, background traffic, and the starting time of each flow. IEEE 802.11p, a well-known communication approach for vehicle-to-vehicle communication, is similar to Wi-Fi. On the other hand, vehicles can also communicate with each other by cellular networks. Therefore, this findings from this study can be used in designing a new MPTCP approach for file transfer and video streaming in vehicular environments.

In [41], Deng et al. compared transmission performances of Wi-Fi and LTE by conducting a real world experiment. A crowed sourced mobile application was used by 750 users over 180 days from 16 different countries in the experiment. The results show that at a considerable amount of time, LTE outperforms Wi-Fi, since Wi-Fi performance is affected by signal quality or user density easily. They also compared the MPTCP performance with TCP performance over Wi-Fi or LTE from 20 different locations of seven cities in USA. For short flows, the faster link of regular TCP outperforms MPTCP performance. To shorten the transmission time for small size data, it is critical for MPTCP to choose a proper communication path. The reason is that, as analyzed in [40], for short flows the transmission can be completed soon by a faster regular TCP link. So MPTCP has limited effects on the transmission performance. The situation is similar to MPTCP. To complete transmission quickly, the selection of a proper path is very important. While choosing the primary path is also important for large data flows, applying correct congestion control to increase the bandwidth is more important. In this work, only coupled and decoupled congestion control mechanisms are utilized. On the other hand, on the extreme scenario such as highly heterogeneous networks, the TCP outperforms MPTCP. Therefore, it is very important to make correct decisions about whether to apply MPTCP and which paths to use in MPTCP based on the network environments. Several mobile phone applications are also analyzed to complement flow level analysis.

Han et al. [42] conducted experiments to compare the performance of HTTP and SPDY over MPTCP and SPTCP. The result is that SPDY benefits more from MPTCP. SPDY protocol is developed based on multiplexing technology. However, they also discovered that MPTCP does not always bring performance improvements for mobile web applications. Some browsers such as Chrome perform even worse when using MPTCP at HTTP. In a mobile scenario, the IP address needs to be changed when an end host moves out of the coverage zone of an access point if using SPTCP. The on-going connections would be stopped or reset. The uncompleted data will be discarded in case of a reset. However, MPTCP can solve the disconnection problem in a moving environment [43,44]. On the other hand, the signal strength is unstable in a mobile scenario. Frequent packet losses may occur in this situation, which would result in insufficient bandwidth usage. This is because the congestion window is not able to reach the fully utilized state. In case of MPTCP, this phenomenon can be mitigated by shifting the flows to the paths with stronger signal strength.

## 3. Congestion Control and Scheduler

MPTCP mainly has two important flow managing mechanisms, namely, congestion control and packet scheduler. The congestion control is in charge of controlling induced network load, and to avoid network congestion. The traditional single path TCP utilizes the congestion avoidance algorithm, which includes the slow start scheme and additive increase/multiplicative decrease (AIMD) scheme. The MPTCP congestion control mechanism was designed [45] and standardized [46]. On the other hand, the scheduler is responsible for distributing data over the multiple paths. A proper scheduler can improve the goodput (application-level throughput). However, unlike MPTCP congestion control, the scheduler has not been standardized yet. The most commonly used schedulers are round robin (RR) scheduler and shortest smoothed RTT (SRTT). The Linux kernel MPTCP implementation [47] scheduler is based on SRTT. Many studies regarding MPTCP congestion control and scheduler have been conducted in recent years. Meanwhile, there are also some studies that combine MPTCP congestion control and scheduler together to improve transmission performance. For example, Tang et al. [48] proposed a solution to improve the transmission efficiency of short TCP flows. The main idea is to create a path cluster with similar characters based on RTT. Meanwhile, some research efforts that prevent long flows from preempting resources have been made. In the following section, we introduce the studies regarding MPTCP congestion control and scheduler.

### 3.1. Congestion Control

#### 3.1.1. Congestion Control with Predefined Rules

Many fundamental MPTCP congestion control mechanisms have been proposed in the past few years, including linked increases algorithm (LIA) [46], opportunistic linked-increases congestion control algorithm (OLIA) [32], weighted vegas (wVegas) [49], and so forth. Among these MPTCP congestion control mechanisms, LIA is the standard coupled MPTCP congestion control mechanism. It couples the congestion windows of all the subflows and controls the total window size as one TCP connection, hence achieving the goal of TCP fairness. This mechanism also guarantees that the total throughput of all subflows is not less than the throughput as a TCP run on the best path. On the other hand, non-coupled is a simple extension of TCP to multiple paths. However, due to its aggressiveness usage of resources on the shared network, it causes a TCP unfairness problem. Congestion control mechanisms for MPTCP are mainly divided into loss-based congestion control (CC) and delay-based congestion control. The loss-based CC triggers an event to adjust the window size based on packet loss, while the delay-based CC adjusts window size based on the packet transmission delay. The LIA not only takes into account the packet loss rate for the congestion control like the classic Reno [50] does, but also considers the round trip time (RTT) for congestion window adjustment. However, bufferbloat [51] will occur in heterogeneous networks for LIA, which would result in delayed packet delivery to the application layer. Cao et al. [49] proposed wVegas, which utilizes packet queuing delay to adjust congestion window size. By considering the delay more accurately in the congestion control, wVegas is more suitable for dynamic network environments. Moreover, it also occupies fewer link buffers and is able to reduce losses compared with loss-based algorithms. Smiliar to the way implemented by compound TCP, it is also being considered as a promising way by combining wVegas and LIA.

As mentioned earlier in this paper, one of the main goals of MPTCP is to achieve TCP friendliness. One way to reach this goal is to couple different paths in a shared bottleneck. Many studies have been conducted to optimize congestion control on the premise of achieving TCP friendliness. Honda et al. [52] presented a TCP-friendly approach for multipath transport protocol, namely bidimensional-probe multipath congestion control (BMC). It includes an aggressiveness manager and proportion manager. Aggressiveness manager is designed to ensure subflows throughput. In order to maximize the entire connection throughput, the proportion manager is for adjusting the weight of different subflows. In [53], Hassayoun et al. present a congestion control mechanism, called dynamic window coupling (DWC). DWC is able to detect shared bottlenecks by loss and delay signals. Then it couples or decouples the subflows depending on the detection results. Furthermore, DWC is able to detect the shifting of bottlenecks on various network environments. The DWC improves throughput by aggregating across distinct bottlenecks and achieves fairness among TCP flows.

Singh et al. [54] compared different congestion control variants of MPTCP, namely, NewReno, LIA, and DWC, in different scenarios. They further developed some DWC extensions to detect the subflows with the same bottleneck (multiple subflows are transmitting through the same network path that becomes the bottleneck of the network). Among the extensions, Delay-Delay variant has the highest efficiency in detecting bottlenecks because it can avoid active queue management problem. Delay-Delay variant utilizes a delay congestion event both in triggering congestion inspection, and grouping subflows with the same bottleneck.

Li et al. [55] proposed DBCCA to optimize the transmission performance in heterogeneous networks. The DBCCA is a delay-based approach that consists of two phases. In the first phase, DBCCA minimizes the delay gap of different paths and obtains a rate distribution vector. In the second phase, the congestion window is adjusted based on the rate distribution vector acquired in the first phase and RTT. DBCCA also shifts traffic to paths with lower losses to improve overall throughput in heterogeneous networks.

Zhou et al. [56] proposed CWA-MPTCP, a congestion control algorithm for MPTCP. CWA-MPTCP takes goodput as an important metric to adjust the congestion window. Since packets have a fixed sequence in TCP transmission, the delay difference of the paths would result in out-of-order packets arrival in receiver buffer, which will reduce the goodput. This is because the out-of-order packets need to take some time to be reordered in receiver buffer. CWA-MPTCP aims to improve the throughput of MPTCP in a condition where the delay difference of different paths is large.

#### 3.1.2. Congestion Control with AI

Artificial intelligence (AI) has attracted people’s attention and has changed our lives in many aspects, such as health care, manufacturing, navigation, social media, etc. Meanwhile, the above-mentioned conventional MPTCP congestion control algorithm only employ coarse-grained pre-defined rules. Therefore it has some limitations, such as the difficulty in adapting to environmental changes, the bufferbloat problem in heterogeneous networks, underutilization of bandwidth, etc. Hence it is important to apply AI in MPTCP studies. Some studies apply AI technology in multipath TCP congestion control to tune the sender rate dynamically according to the surrounding environment.

In [57], a deep reinforcement learning (DRL)-based framework, namely DRL-CC, is proposed. DRL-CC is an experience-driven MPTCP congestion control algorithm. It mainly consists of two processing steps. Firstly, in order to simplify the sophisticated network scenario, all of the possible states of active flows are processed by a representation network to generate a representation. Variable input is possible by using long short term memory (LSTM). Then the representation is fed into the actor-critic networks to derive an action for congestion control. Continuous control is realized by integrating the LSTM into an actor-critic network framework, and thus dynamic and sophisticated state spaces can be handled. Besides deep reinforcement learning (DRL), the characteristic of centralized control of a software-defined network (SDN) is also deemed to be a solution to MPTCP studies. A model free SDN-based framework, called FNDRL-CC, is proposed in [58]. The major components of FNDRL-CC include a representation network, Actor-Critic, and fuzzy normalized function. The fuzzy normalized function is for solving high dimensional problems. The representation is utilized to train the actor and critic. Then the Actor-Critic network together with the representation network provide a congestion control action to the target flow.

To deal with the diversity paths of the heterogeneous networks, Li et al. [59] proposed a learning-based approach, called SmartCC. The sender learns the congestion control rules by observing environments and conducts the congestion control across subflows. In SmartCC, the training part and execution part are decoupled. The SmartCC contains three stages. At the bootstrapping stage, the existing MPTCP congestion control rules are utilized until the state is found in the rule table. If the states have not been optimized yet at the early stage, the e-greedy method is adopted. At the offline training stage, historical experiences are applied to train a congestion control rules by a trainer. A hierarchical tile coding is adopted to map the continuous CC states into discrete state. At the online decision stage, the agent synchronizes with the trainer and makes online decisions. Mai et al. [60] utilized reinforcement learning and cross layer information in a congestion control problem for satellite communications.

Named data networking (NDN) is a receiver driven transport architecture. Unlike the commonly used IP address-based routing, NDN has the characteristic of name-based routing, and source uncontrollability. Liu et al. [61] proposed ACCP in named data networks. The ACCP has two phases. First, the source of congestion for each node is predicted by the deep learning-based model. Second, the network congestion level is estimated based on the result of the first phase. The congestion control of entire network would be conducted according to the result of the second phase. ACCP performs better than CHoPCoP [62] and ICP [63] in terms of many aspects such as losses. Table 2 summarizes the studies on MPTCP congestion control.

#### 3.1.3. Lesson Learned

In this subsection, we present some multipath congestion control mechanisms. Each mechanism has its own characteristic and is designed for a specific purpose. For example, LIA is proposed for solving the TCP-friendliness problem. LIA is supposed to solve the problem that a simple extension of TCP to multipath by applying AIMD on each path independently would result in TCP unfairness. As LIA and wVegas are extensions of the single-path TCP congestion control algorithm, they inherit some characteristics of single-path TCP. When the network condition is stable, a simple extension of single-path TCP to multiple paths can be an acceptable way. However, these methods have limitations in irregular network conditions such as lossy networks or heterogeneous networks.

In order to overcome these limitations, some algorithms have been proposed. For example, DBCCA is designed for lossy heterogeneous network environments. Although these approaches show their advantages under certain network conditions, more extensive experiments are still needed to verify the effectiveness. On the other hand, new architectural design that combined with other techniques such as SDN should be considered to satisfy the goals.

Due to the constant development of new technologies such as collaborative autonomous driving or remote surgery, the expectations for network performance are also rising. Especially, for vehicular networks in which the network topology and network condition are constantly changing due to the movement of vehicles. In this case, the single path TCP extensions and the existing algorithms designed for other purposes cannot meet the performance requirements.

To overcome the limitation, some learning-based congestion control methods are utilized to dynamically adjust the congestion control rules according to the change of network topologies. The learning-based approach has achieved some promising results. However, there are some issues that require further discussions. First, the machine learning-based approach requires some time to train the model. Second, more efforts need to be taken on the training accuracy. In a complex scenario, it is challenging to accurately perceive the environment and therefore the information acquired at each agent is inaccurate. The machine learning process should fully address these inaccuracies in the input, process, and output steps of the training. Third, TCP-friendliness is not considered adequately in some learning-based approaches.

Some issues should be further discussed in future work. First, AI-based congestion control algorithms need to be tested under real-world network scenarios to verify their effectiveness. Second, AI-based schedulers need to be further investigated to improve the efficiency of multipath congestion control by jointly utilizing a pre-trained model or proper mechanisms according to the scenarios. Third, it is also a promising approach to consider combining different congestion control methods that complement each other. Third, TCP-friendliness issues should be discussed more carefully in learning-based congestion control algorithms. Last but not the least, it is also important to discuss the energy efficiency problem of congestion control algorithms. We will introduce some energy efficient MPTCP variants in the next section.

### 3.2. Scheduler

#### 3.2.1. Pre-Defined Scheduler

**Figure 3:**
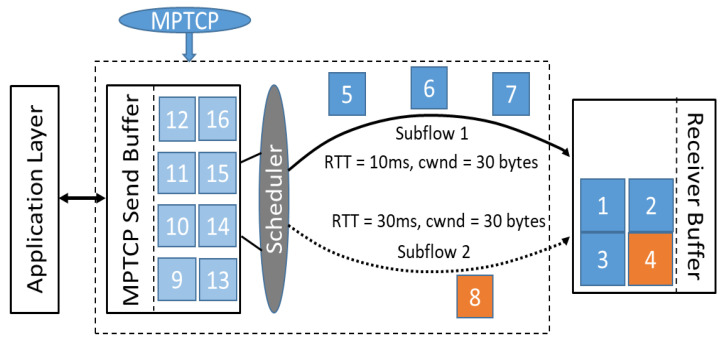
Multipath TCP scheduler.

As mentioned above, scheduler is another important part that greatly influences MPTCP performance. Generally, a scheduler has to consider two main constraints, namely, head-of-line (HoL) blocking [64] and receive-window limitations. Round-Robin (RR) and Lowest-RTT-First (LowRTT) [65] are well-used schedulers. The RR scheduler distributes packets through every active path in a circular order, regardless of whether there are severe latency differences among different paths. On the other hand, to mitigate the effects of delay difference in heterogeneous networks, LowRTT prioritizes fast flows then relatively slow flows until the congestion window is filled up. For example, as shown in Figure 3, the MPTCP scheduler sends three packets on the fast subflow 1 while only sending one on the slow subflow 2.

There have been many studies in regard to the scheduler. In [66], Frommgen et al. proposed a scheduler called ReMP. The ReMP duplicates packets over all subflows in order to obtain reliability. Experiments show that ReMP halves the average round-trip time. However, this way may cause additional overhead. MPTCP implementation is available in Linux kernel [47]. The intelligent Linux-MPTCP scheduler [67] is implemented in the MPTCP Linux kernel. The scheduler first estimates the available bandwidth of each subflow, then distributes data packets based on the estimation proportionally. In addition, packets to be sent can be selected to transmit from the shared sending buffer. However, this scheduler is fragile in lossy networks.

Completing all of the subflows transmissions at the same time will benefit the entire transmission for MPTCP. It is considered that the MPTCP’s default scheduler is suboptimal because the default scheduler makes decisions based solely on RTT. It could cause congestion at a high speed path due to injecting too many packets into it, which would result in bufferbloat because of out-of-order packets. Therefore, a scheduler should also consider the congestion situation of paths while scheduling data packets across different subflows. In [68], Yang et al. proposed a scheduler that first estimates the available bandwidth of the paths and then decides the scheduling policy based on the path capacity of the subflows. The path utilization rate can be maximized by keeping the paths longer at a saturation point.

In order to complete all the subflow transmissions at the same time in heterogeneous networks, Raiciu et al. [65] introduced a RTT-based scheduler extension, namely, opportunistic retransmission and penalization. The main purpose of this extension is for compensating the delay differences on different paths. The segment that causes HoL will be resent across other available flows in RP. Similar to RP, CMT-SCTP [69] triggers chunk rescheduling when the transmission is stalled in asymmetric paths. Similarly, Oliveria et al. [70] proposed an algorithm, called bufferbloat mitigation (BM). The BM is for suppressing the RTT by limiting the amount of transmission data.

Besides the above-mentioned studies, there are some efforts on reducing the delay difference and data-sorting cost. In [71], Hasegawa et al. proposed a data distribution method called ATLB. ATLB consists of three parts, namely, data distribution, path-failure detection, and recovery mechanism. The data distribution method is designed for reducing the data-sorting cost of the receiver buffer. The path-failure detection and recovery mechanism is for preventing transmission stalling. In [72], Guo et al. proposed a scheduler called decoupled multipath scheduler (DEMS). The DEMS aims at improving data chunk download efficiency over multiple paths. DEMS is robust to diverse network conditions, especially for fetching medium size files and small size web pages.

A blocking estimation-based method (BLEST) was proposed in [73]. BLEST aims to minimize HoL blocking at the receiver side in heterogeneous networks. MPTCP tackles the HoL blocking problem by penalizing the utilization of longer paths and increasing the receiving buffer sizes. However, this is considered to be suboptimal. Therefore, the BLEST scheduler minimizes the HoL blocking by reducing the number of unnecessary retransmissions.

Another approach to mitigate bufferbloat in heterogeneous networks is to ensure in-order packet arrivals. WestwoodSCTP [33] sorts each transmission path, and transmits the packets in descending order of rank. However, it still cannot reach the goal when significant different latencies exist among different paths. To overcome this significant latency gap, Mirani et al. [74] proposed the FPS mechanism, which not only estimates the arrival time of packets on the transmitting path, but also estimates the data delivery time on the other paths, realizing the data arrivals at the same time on all paths. However, FPS only considers RTT as an estimation metric and does not consider lossy networks. Instead of only utilizing RTT, Xue et al. [75] proposed DPSAF, dynamic packet scheduling, and adjusting with feedback. DPSAF consider the loss rate in packet scheduling. DPSAF is able to conduct fine-grained scheduling based on the amount of estimated data which is obtained by applying maximum likelihood estimation on all transmitting path and therefore is capable of responding quickly to network changes due to frequent feedback receptions. Ni et al. [76] proposed a packet scheduling mechanism, called F2P-DPS in lossy networks. In addition to RTT and loss rate, it also takes congestion window size (cwnd) into consideration. F2P-DPS models the TCP’s behavior to calculate the latency on the scheduling path. Meanwhile, the states of other transmitting paths will also be estimated. F2P-DPS outperforms FPS in throughput and in-order packets arrival.

Since delay is more critical for small size flows, in order to complete the transmission as quickly as possible, Hwang et al. [77] proposed a scheduler which stops the slow path temporarily if there are large delay gaps between different transmission paths. A generic modular scheduler framework was designed in [78]. This schedular is able to change the data distribution over the subflows easily. The framework is utilized for testing different schedulers of multipath TCP. In [79], Chayyasith et al. proposed a delay-aware based path scheduling scheme on asymmetric paths for multipath transmissions. The proposed scheme improves the link utilization rate and in-order packets arrival rate in the receiver buffer. However, the scheme currently only supports single TCP flow, and further studies are required to support multiple flows and assure fairness among flows.

#### 3.2.2. Scheduler with AI

Many studies applied AI technology to a multipath TCP scheduler to refine the scheduling policy dynamically in accordance with its surrounding environments. In [80], Zhang et al. proposed a RL-based scheduler, namely, ReLes, to generate packet scheduling policy dynamically. Different from the traditional pre-defined fixed policies, ReLes is able to adjust the scheduling policy dynamically based on network environments. The scheduler takes the observations of the environments, such as RTT and throughput measurement, as an input, and generates a policy to allocate data on subflows as an output by using a neural network. The training part of the neural network is conducted in an offline way.

In MPTCP networks, choosing the best path among all available paths is also challenging because it depends on the loss rates, RTTs, and data sizes. In most cases, the path state information is not available a priori, and the loss rates and RTTs will change over time. To overcome the limitation, Luo et al. [81] proposed a method based on RL with deep Q network (DQN). The method is able to play a role in choosing the best path in MPTCP heterogeneous networks. The method can be enhanced by taking more network metrics into consideration to improve the selection accuracy.

Rosello [82] applied deep reinforcement learning to improve packet scheduling. Instead of using fixed parameters such as SRTT and congestion window for characterizing the performance of an access path, a deep reinforcement learning agent is utilized to learn the strategy for distributing a packet optimally, thereby increasing the aggregated throughput of the multiple flows. The agent has two main phases, namely, the feedforward and backpropagation. In the feedforward phase, the agent interacts with the environment and acquires an optimal strategy to distribute the packets. The backpropagation phase is implemented in an offline way, and the deep neural network is updated by the previous experiences at this phase. However, the improvement is not big. Khalili et al. [83] mentioned that MPTCP can respond to network changes by performing congestion balancing across different paths without prior knowledge. Table 3 summarizes the studies on the MPTCP scheduler.

#### 3.2.3. Lesson Learned

In this subsection, we introduce some multipath TCP schedulers. We categorized the schedulers into four groups here, including simple scheduler, path quality estimation-based scheduler, delay difference-based scheduler, and learning-based scheduler.

Simple schedulers, such as RR and LowRTT, simply distribute packets in a circular order or based on RTT. However, these methods have limitations in irregular network conditions such as lossy networks or heterogeneous networks. In order to sufficiently utilize the network resources by keeping the paths longer at a saturation point, the path quality estimation-based scheduler estimates the bandwidth or transmission time on all available paths before distributing data. However, the path estimation-based schedulers face some challenges if the network condition changes very quickly. This is because the estimated path states will occur deviation as time goes on. Although some efforts have been made to discover network changes and make adjustment on the scheduling strategy accordingly, it is still unable to predict the network changes in advance or implement scheduling synchronously with network changes by a path quality estimation-based scheduler. On the other hand, the delay difference-based schedulers are for minimizing the delay gap of different paths. By doing this, the data packets that transmitted on different paths can arrive at the receiver buffer simultaneously, avoiding packet reordering. However, this method has the disadvantage of sacrificing the bandwidth of fast paths. Meanwhile, in order to solve the delay difference problem for short flows, there is the possibility of disabling the slow path when the delay difference between different paths is huge. However, it will result in a lower usage rate of multiple paths.

The aforementioned approaches are pre-determined schedulers and are hard to satisfy the performance requirements of recent new technologies such as vehicular networks. In order to meet the requirements of highly mobile networks, some learning-based schedulers are utilized to dynamically adjust the scheduling policy according to the changing network environments. The learning-based approaches have explored a new area for MPTCP scheduling. However, they also have some problems. First, the learning-based approaches need some time to train the model before convergence. Second, the performance improvement is not significant when applying deep reinforcement learning in some scenarios. Furthermore, sometimes a learning-based scheduler is outperformed by fundamental schedulers under some settings.

For future works, firstly, it is a promising way to consider a combined design, including the combination of scheduler with congestion control. Second, while most AI-based schedulers are trained and tested based on simulation data, it is urgent to test their performance in real-world network scenarios to verify their effectiveness. It is also necessary to further review the state and reward in order to ensure the validity of the learning model. Thirdly, it could be a solution to overcome the training delay by combining an AI-based scheduler with a pre-trained model or proper mechanisms according to the scenarios. Last, a study of energy efficiency problem on the scheduler design could also be an important future research topic.

## 4. Cross Layer MPTCP, MPTCP with SDN, and Energy Efficient MPTCP

With the improvement of congestion control and the scheduler, MPTCP was improved significantly. It is believed that the performance can be enhanced by increasing the granularity of control. In order to achieve a fine-grained control, MPTCP conducts cross-layer management by considering the information of other layers. For example, MPTCP takes how the network interfaces are used into consideration. Similarly, the characteristics of the centralized control of SDN is also considered to be a promising solution to refine MPTCP control. Meanwhile, there is also one concern for using MPTCP, specifically, the high energy consumption for maintaining multiple active interfaces. However, the congested flow can be shifted to a less congested one without breaking the connection in MPTCP. By doing this, the completion time can be reduced, and thus energy can be saved. In the following section, we introduce several studies that are related to cross layer MPTCP, MPTCP combined with SDN, and MPTCP energy efficiency.

### 4.1. Cross Layer MPTCP Approach

In [84], Withnell et al. presented a MPTCP cross-layer context aware approach called MPTCP-CA, to manage and utilize network resources. MPTCP-CA cooperates with other layers to perceive the entire network states, based on which subflows are created or destroyed. They also provide a guidance for how to utilize network interfaces. The architecture of the MPTCP-CA consists of kernel space and user space. Path manager resides in the kernel space, and triggers a communication with user space applications when a MPTCP connection starts. The core logic of the path manager is implemented at the user space in order to ensure configurability and flexibility. At the same time, it is able to have the path manager extended with an API. In [85], Lim et al. proposed a cross layer MPTCP path managers, namely, MPTCP-MA. They utilize the link quality information of MAC layer to help to decide how to manage subflows. More specifically, subflows are decided either suspending or releasing according to the MAC Layer information. For example, if the number of MAC-Layer frame retransmissions becomes larger than the number of successful frame transmissions at the low PHY data rate, then it will be notified that the Wi-Fi subflow is unavailable and set to inactive.

In [86], Coudron et al. proposed a MPTCP cross layer cooperation module for cloud networks, namely, augmented MPTCP (A-MPTCP). The A-MPTCP is able to fold the transmission time with the assistance of location/identifier separation protocol (LISP). In A-MPTCP, the information from the user space is used to inform the path manager about the available paths information of cloud environments.

### 4.2. MPTCP with SDN

Pol et al. [87] proposed the first work about combining MPTCP and OpenFlow. OpenFlow is an enabler of SDN that can conduct traffic engineering within the network, such as identifying the network topology. SDN technology can also be used to select the best path for the first subflow [88].

Equal-cost multipath protocol (ECMP) [89] can divide a data stream and send across different paths. However, it only forwards the flows through the shortest path and hence it may forward the subflows through the same path. In [90], Sandri et al. proposed a method, called multiflow, by combining MPTCP and OpenFlow together. Unlike ECMP, the main purpose of this method is splitting data stream over different paths of a connection in shared bottlenecks. Multiflow is able to achieve fine-grained control of subflows by dividing MPTCP subflows for OpenFlow networks. However, the throughput of the MPTCP connection increases only when enough disjointed paths exist.

Resource pooling principle is also discussed in the MPTCP design. In [91], a SDN-based MPTCP, namely, called S-MPTCP, is proposed for the efficient utilization of bandwidth and fair resource allocation by utilizing global network information.

Many studies are only devoted to improving the transmission performance by MPTCP, while neglecting the price cost. Gao et al. [92] proposed SOS-MPTCP in a software defined wireless network (SDWN), which aims to balance the performance and cost properly.

### 4.3. Energy Efficient MPTCP

An energy-aware congestion control algorithm, namely, ecMTCP, is proposed in [93]. The ecMTCP mainly consists of two parts. The contribution of ecMTCP is to achieve energy-savings by shifting the traffic from the most congested path to a less loaded path.

However, if the energy consumption is the most important criterion for MPTCP transmissions, the goal can be achieved by sacrificing other network metrics such as throughput and latency slightly. In [94], Pluntke et al. proposed a multipath scheduler using MPTCP to improve energy efficiency. They achieve energy efficiency with slight scarification of throughput. The scheduler takes the energy model of different radio interfaces, communication history of the device user, and application model into consideration and switches between multiple paths properly to save energy. Similarly, Peng et al. [95] proposed an algorithm that takes a tradeoff between the throughput and energy consumption for MPTCP by shifting a traffic to an energy efficient path.

Likewise, an energy-aware MPTCP, namely, eMPTCP, was designed in [96]. This approach combines power-aware subflow management and delayed subflow establishment to reduce power consumption with a small negative impact on latency. The power-aware subflow management is for selecting path dynamically to maximize energy efficiency. The delayed subflow establishment is for avoiding power consumption for small size file transfers. Since web browsing applications are delay-sensitive, browsers can determine whether to enable MPTCP based on whether the secondary path supports a small latency.

## 5. MPTCP in Vehicular Networks

Vehicular network is an important part of intelligent traffic systems (ITS). Vehicles can communicate with cars, roadside infrastructure, and pedestrians, and drivers can exchange information more conveniently and efficiently through vehicular networks. However, due to the constant mobility of vehicles, the vehicular network has the feature of dynamic topology and various node densities, which makes it challenging to provide a stable and robust connection between vehicles. At the same time, most of the vehicular applications have stringent requirements on the response delay and reliability. There are some efforts that improve vehicular networks performance based on single path TCP. Bechler et al. in [97] proposed a mobile control transport protocol (MCTP) in VANETs, which is a proxy-based communication protocol. Vehicles communicate with proxy by MCTP, and proxy communicates with the Internet with TCP. The main idea of the MCTP is to distinguish network problems, such as packet losses, congestion, and network disconnections. MCTP is located between the TCP and IP, so that it can observe the packet flow by utilizing the intermediate systems and underlying protocols. In a conventional handover approach, the network performance degradation occurs, due to the change in IP address. Since the TCP connection will be released and reestablished. This would result in the CWND falling into the initial value. Ran Duo et al. in [98] proposed a SDN-based handover scheme in vehicular networks. To overcome the degradation, they utilized SDN technology to map the old connections information to the new connection, ensuring a seamless handover.

**Figure 4:**
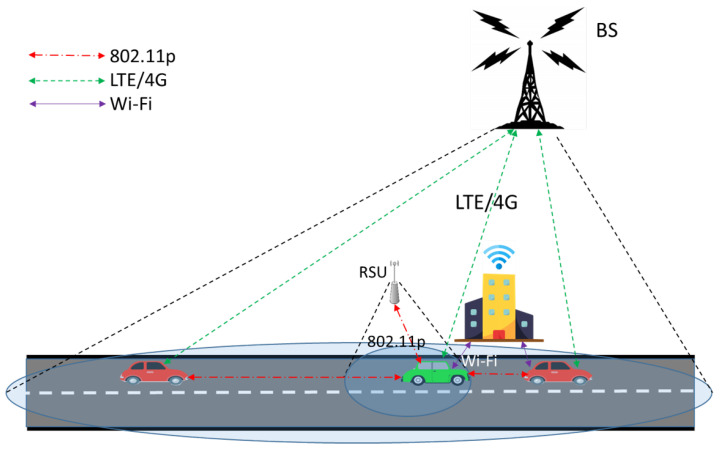
MPTCP in vehicular networks.

Nowadays, vehicles could have different types of communication interfaces. There are also many types of wireless communication technologies such as IEEE 802.11p, cellular communication, and mmWave, available for vehicular networks. Among them IEEE 802.11p is the default standard communication technology for vehicle-to-vehicle communication. To improve the connection robustness and transmission efficiency in vehicular networks, MPTCP is considered to be a promising approach. MPTCP can facilitate mobility [43,44] compared to single path TCP. As shown in Figure 4, MPTCP has the ability to improve vehicular network throughput by combining these wireless communication technologies, and to provide robust connectivity [99] by implementing handover between different subflows when interruption incurs. For example, in Figure 4, the green car can communicate with RSU and adjacent cars by IEEE 802.11p, base station (BS) by cellular, and building by Wi-Fi simultaneously in one TCP connection. However, MPTCP currently does not perform well under very high velocities in vehicular environments.

There are many studies that discuss MPTCP in vehicular networks to improve the transmission rate and reduce latency. In the following, we introduce some research efforts on MPTCP in vehicular networks. We summarize the studies in Table 4.

### 5.1. Existing Studies on MPTCP in Vehicular Environment

Nigel et al. [100] conducted some experiments and showed that MPTCP can improve the overall communication throughput in vehicle-to-infrastructure (V2I) environments when the multiple paths have similar characteristics. MPTCP-enabled vehicles create multiple flows by triggering a link with road side unit (RSU) with IEEE 802.11p while connected to the base station with cellular communications. The IEEE 802.11p link will break when vehicle moves out of the communication range of the RSU. Despite retransmission being required when the link disruption occurs, the performance of using MPTCP is still larger than the conventional single path TCP. In [101], Zhu et al. made efforts on improving the stability of multipath TCP transmissions. A vehicle detects whether it is in the communication range of RSU. When the vehicle is in the communication range of RSU, it communicates with the server through both the RSUs and cellular base station concurrently. The vehicle transmits data only with the stable interface (here is the cellular interface) when it moves out of the transmission range of the RSU. In order to improve the transmission performance in heterogeneous networks, Cloud et al. [102] first conducted an empirical measurement for the simultaneous use of three different communication approaches in heterogeneous network environments. Then they propose a MPTCP with networking coding (MPTCP/NC) protocol. The advantage of MPTCP/NC is maintaining throughput in lossy networks by recovering lost packets with some redundancies. In [103], Bai et al. also proposed a network coding-based MPTCP (NCMPTCP) for vehicular ad hoc networks (VANETs) to handle the communication bottle neck incurred from vehicle mobility.

Zhao et al. [104] introduced Q-Learning based MPTCP to schedule the path and reduce the buffer blocking in heterogeneous networks. In [105], Nan et al. proposed PTLIA, a congestion control algorithm based on message priority and throughput estimation, to improve the transmission performance. For example, the security messages have a high priority, and therefore they are needed to be sent prior to other messages.

Dong et al. [106] introduced a concurrent multipath transmission scheme for high speed trains. They implemented an experiment that aggregated six available links to provide bandwidth to the onboard devices. Lee et al. [107] discussed a collaborative MPTCP architecture to achieve fine-grained control. In addition to protocol layer information, such as RTT, and congestion window, the device status and user preference are also considered to achieve an efficient transmission. For example, the decision of whether to turn on or off the MPTCP when commuting or reading in library based on user preference. In [108], Rene et al. proposed a mechanism that combines MPTCP with deep packet inspection (DPI). The mechanism utilizes selective discarding on the sender side and partial reliability on the receiver side to reduce the waiting time at buffer.

There are also studies that apply MPTCP in unmanned aerial vehicles (UAVs). Chirwa et al. in [109] proposed an unmanned aerial system (UAS), which utilizes multiple cooperating UAVs. UAS also faces problems of frequent disconnection and high packet loss rates due to the mobility of UAVs. To improve performance, the UAS utilizes MPTCP to ensure robust connection and seamless handover on heterogeneous paths. They set up a mathematical model to match the UAV interface type to transmission requirements, and thus achieve optimal traffic allocation. For example, they set larger window openings to appropriate interfaces. Jung et al. in [110] proposed an adaptive offloading architecture with MPTCP for UAVs. The architecture consists of three modules, namely, the response time prediction module, task offloading module, and remote task execution module. The response time prediction module and task offloading module are embedded in UAVs, while the remote task execution module is in the ground control system (GCS). The task offloading module communicates with the remote task execution module by MPTCP. Considered the high mobility feature of UAVs, the default scheduler of MPTCP is modified by considering the network handoff time.

### 5.2. Lesson Learned

In this section, we introduced some studies on MPTCP for vehicular networks. MPTCP is able to improve overall throughput and stability under certain network conditions in V2I. However, packet losses occur frequently when vehicles disconnect with infrastructure because of the frequent movement of vehicles. The application-level delay also increases due to retransmissions. In addition, the communication bottleneck problem is also pointed out in heterogeneous networks. It implies that although MPTCP has a potential to play a role in vehicular networks, some further improvements are required. The MPTCP in vehicular networks is expected to bring the following benefits.

- Higher bandwidth utilization rate: MPTCP could realize better utilization of network bandwidth by conducting transmissions through multiple paths;
- Better performance: It is possible for MPTCP to achieve more stable transmissions by shifting between different subflows. When interruption incurs on one flow, the on-going transmission data can be shifted to other flows without reset or changing the IP address;
- More powerful applications: Since MPTCP can support a higher performance than the conventional single flow TCP, MPTCP could enable some new applications such as real time high definition video streaming.

Existing studies show that we can improve the performance of MPTCP by leveraging other techniques. Network coding-based MPTCP approaches are able to maintain a high throughput in lossy networks by recovering lost packets. However, the network coding based approaches are only evaluated with theoretical analysis or simulations, and are not verified by real-world experiments. The Q-learning based approach is able to reduce buffer blocking in heterogeneous networks. However, the convergence of the model needs some time. It would be more beneficial if a pre-trained model is available.

In addition to only considering the metrics of a transmission layer, other factors are also taken into consideration to collaboratively satisfy the design goals. For example, the message priority, device status, and user preference are utilized in some studies. However, these studies stay in the proposal stage or simple simulation stage. Performance metrics such as throughput and energy consumption need to be further investigated by real-world experiments. Other factors that influence network performance also need to be further investigated.

Some important future research topics are as follows. First, it is urgent to design an efficient MPTCP architecture that addresses the characteristics of vehicular networks by considering more factors in order to satisfy user requirements in various scenarios. Second, AI-based solutions should be further investigated. Third, in order to enable an efficient MPTCP transmission, a support from the underlying routing layer is mandatory. Choosing proper routes to finish transmissions efficiently before topology change would be a promising way. Therefore, more research efforts are required to enable an efficient integration of MPTCP and multipath routing. Lastly, context-aware approaches should be further discussed as the scheduling of multipath transmissions should fully consider the contexts, including user requirements, available network resources, vehicle distributions, vehicle capability, and so forth.

## 6. Mutipath Routing in Vehicular Networks

**Figure 5:**
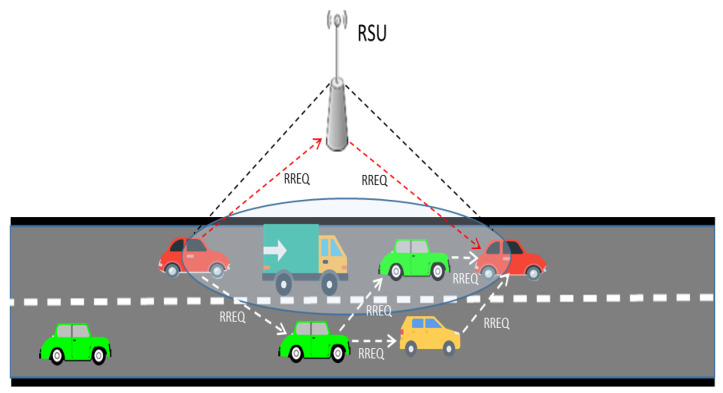
Multipath routing in vehicular networks.

A vehicular ad hoc network has the well-known problems of frequent link disruptions and a high packet loss ratio. Besides MPTCP, multipath routing is considered to be another promising approach that can improve vehicular network performance by establishing multiple routes between vehicles to enhance the connectivity in the network layer. As shown in Figure 5, there are three paths between the two red cars.

There are also studies that combine MPTCP and multipath routing [10,11] to enhance network performance. In [111], Gupta et al. combined the connectivity aware routing (CAR) and MPTCP for file transfer between RSUs and vehicles in multi-hop communications. They also compared the performance of CAR and MPTCP. However, in sparse vehicular network environments, traditional routing protocols cannot perform effectively due to the lack of relay nodes to maintain the connections, which would result in excessive retransmissions. A delay tolerant network (DTN) is considered to be a promising way to solve this problem. Filho et al. [112] conducted a systematic technical survey about the DTN. The VANET/DTN (VDTN) is also included. In the following section, we only give a brief review of traditional multipath routing research efforts. We classify the related studies into five categories, respectively, extensions of ad hoc routing protocols, fault-tolerance and security, cross-layer multipath routing, optimization model, and stochastic model.

### 6.1. Extensions of Ad Hoc Routing Protocols

There are many single path routing protocols such as ad hoc on-demand distance vector (AODV) [113], dynamic source routing (DSR) [114], DSDV (dynamic destination-sequenced distance-vector) [115], and TORA (temporally-ordered routing algorithm) [116]. In order to enhance the connectivity between end hosts and transmission efficiency, many multipath routing protocols that extend AODV and DSR were proposed. For example, ad-hoc on-demand multipath distance vector (AOMDV) [9] and multipath source routing (MSR) [8]. In [117], Wu et al. compared the performance of AOMDV with AODV. AOMDV outperforms AODV in throughput, packet loss rate, and end-to-end delay. Most importantly AOMDV is less influenced by vehicle speed and density compared to AODV. MSR extends DSR’s route discovery and route maintenance mechanism. As part of the maintenance mechanism, a probing mechanism is employed to balance the load between paths based on the measurement of RTT. In [118], based on the advantages of AOMDV, such as fault-tolerant and load balance, an improved AOMDV with speed metric namely S-AOMDV was proposed.

### 6.2. Fault-Tolerance and Security

Path breakage happens frequently in vehicular networks due to vehicle mobility. Meanwhile, VANETs are vulnerable to misbehaving nodes. Therefore, there are numerous studies focusing on fault-tolerant, fast restoration, or security. Wu et al. [119] proposed a fast restoration on-demand multi path routing (FROMR) scheme. FROMR focuses on rapidly building an alternate path when original path breaks. Zhang et al. [120] developed a leaky-path model by evaluating the effect of faults by using statistical and estimation information. In addition, fault-correlation between different paths is also considered in the routing approach. The results demonstrate that the algorithm outperforms the standard flow control approach in terms of network throughput and fairness. Some efforts have been made to handle malicious attack issues. Baskar et al. [121] proposed a multipath routing protocol that considers user QoS requirements and the trust of nodes. At first, the trusted nodes are extracted by collecting opinions from neighboring nodes. Each node is assigned with a trust value. Then, a trusted route is generated by taking into account the route cost metric (delay and so on) and trust value of nodes for route selection.

### 6.3. Optimization Models

Due to the high mobility of VANETs, transmitting videos via multiple paths is able to increase the chance of video delivery. In [122], Salkuyeh et al. minimized the packet loss rate by optimally distributing video packets in multiple routes for supporting real-time video delivery. Quadros et al. [123] have proposed MVIDE, multi-flow-driven video delivery mechanism, for supporting the live streaming of video data in VANETs. MVIDE communicates with underlying routing protocol to select multipath routes considering the joint performance of multiple routes, network dynamics, and QoS requirements. A routing scheme, namely, learning automata-based optimized multipath routing using leapfrog algorithm (LA-MPRLF), was proposed in [124]. First, multiple available paths are determined by practice swarm optimization (PSO). Then several proper paths are selected among the available paths by learning automata. Leapfrog algorithm is employed to handle path disconnections. LA-MPRLF outperforms AOMDV in packet delivery ratio and throughput. It is shown in [124] that extra overhead is inevitable in VANET communication environments due to reasons such as insufficient usage of computed paths. Devangavi et al. [125] proposed a routing method in VANETs. The main effort of this method is to improve the utilization of the paths sufficiently by utilizing as many computed paths as possible. In [126], several independent routes are determined based on requested video size and lifetime.

### 6.4. Cross-Layer Multipath Routing

A cross-layer position-based delay-aware communication protocol called PROMPT is proposed in [127]. PROMPT adopts a position-based routing approach to deal with the mobility problem in vehicular environments. The vehicles exchange information with each other by sending beacons to estimate the delay and select the minimum delay path. In PROMPT, the routes are expressed as a sequence of street and direction. The relay node is chosen based on the receiver-based MAC channel contention approach, which is related to the distance and direction of the relay nodes from the transmitter. In [128], Rehman et al. proposed a cross layer routing scheme for VANETs. This scheme gathers queuing information of all neighboring nodes and channel information to make optimal routing decision. The method of selecting neighboring nodes set is also discussed in this work. A cross-layer multi-path routing (CLMR) protocol among application, network, media access control (MAC), and the physical layer was proposed [129]. Redundant array inexpensive disks (RAID) technology is utilized to minimize the retransmissions. Chen et al. [130] proposed R-S-AOMDV based on an improvement of AOMDV. In addition to the hop count, R-S-AOMDV considers the link quality and vehicle movement in the route selection.

### 6.5. Stochastic Models

In wireless networks, multiple paths are influenced by each other due to mutual interferences or path coupling. In [7], Huang et al. proved that node-disjoint paths are more efficient than single paths when path-coupling and source-destination distance are chosen properly. To reduce the route coupling, Bandyopadhyay et al. [131] presented that the performance of multipath routing improves when using directional antenna on wireless environments. In [132], Sharma et al. proposed a routing protocol called multipath reliable range node selection distance vector (MRRNSDV). MRRNSDV considers that the data should be transferred through the routes as soon as possible. Therefore, MRRNSDV comes up with a concept, called reliable node, which is a node neither too close nor too far from sender nodes. By selecting a closer node, it will increase the number of hops. In contrast, if selecting a farther node, it will make the route unreliable. In [133], map-based multipath routing protocol (MBMPR) was proposed. MBMPR utilize a GPS, digital map, and sensors to provide supplementary information to vehicles. With the additional information, it discovers the best path and an alternate path by the Dijikstra algorithm. VANETs have the characteristic of intermittent connectivity. Named data networking (NDN) is considered to be able to solve such problems. A multi-hop routing approach was proposed for NDN in [134] where the authors show their approach can achieve faster data delivery with less network resources.

## 7. Open Research Problem

The direct use of conventional TCP in vehicular networks faces some problems. Since TCP is connection-oriented, the constant movement of vehicles would result in frequent link disconnections which is the source of excessive retransmissions. MPTCP also faces a similar problem. Especially when a distributed vehicular communication technology, such as IEEE 802.11p, is used, vehicles face frequent link disconnections due to vehicular movement. One of the advantages of MPTCP is that it can transmit data through stable communication paths when a path is detected as vulnerable. Therefore, it is necessary to understand the performance difference between TCP and MPTCP under various conditions. This can shed a light for future studies. Vehicles can make an optimal decision by deciding how to utilize MPTCP functions based on prior knowledge.

Likewise, the studies of congestion control and scheduler that we reviewed in this paper can also provide a reference to future studies on vehicular networks. Since the network environment in vehicular networks is constantly changing, there is a need to dynamically choose the congestion control and scheduling mechanism properly according to the network conditions. The knowledge can be utilized in the development of vehicular system applications. Although there are some studies that apply AI technology to dynamically adjust the congestion control and scheduling policies, efficiency and accuracy still need to be further improved. For further development, the design of congestion control and scheduling mechanism should consider vehicle mobility and communication capacity, thus optimizing the overall transmission performance. To achieve this goal, the underlying layer can be utilized to help in collecting information.

On the other hand, technology such as SDN has been utilized in the vehicular networks to improve the performance [98]. The SDN technology can be also expected to be utilized for MPTCP in vehicular networks. In order to enable efficient transmissions and robust connectivity in the dynamic and complex vehicular environments, the limited communication resources are needed to be utilized efficiently by MPTCP and multipath routing approaches, which opens up many interesting research topics as the following.

- MPTCP architecture for vehicular IoT applications: There is existing MPTCP architectural study. However, collaborative architecture design for MPTCP would be a continual research topic due to its capability to provide fine-grained control. New applications that cope with improved architecture need to be developed;
- MPTCP resource allocation with AI: The overall system transmission performance depends on the allocation of limited bandwidth resources. Conventional optimization approaches face challenges in solving the resource allocation problem since the optimization-based approach requires a precise prediction of future values of user demands. However, the vehicular environment is very complex and dynamic. Deep reinforcement learning and neural network approaches have the capability to solve the complex problem by dynamically adjusting their policy. A DRL-based MPTCP approach is considered to be a promising way to solve resource allocation problem in vehicular environments;
- Integration of MPTCP and multipath routing: In a highly dynamic environment, it is very difficult to find a robust communication path. MPTCP approaches that collaborate with multipath routing to provide more robust TCP paths that need to be further explored;
- Joint optimization of congestion control algorithm and scheduler: Scheduler and congestion control are the two main components of MPTCP. An efficient integration of these two mechanisms could achieve a better performance;
- Energy efficiency: Some user devices, such as mobile phones, are sensitive to energy consumption. In some studies, the energy efficiency is achieved by sacrificing some other network metrics. A better solution to improve energy efficiency while ensuring other QoS requirements should be discussed;
- Context-aware MPTCP vehicular system: The design of MPTCP vehicular networks should fully consider the vehicular environments, such as vehicle density, vehicle mobility, vehicle capability, and vehicle applications. Since the vehicle connection states change frequently, it is also a promising way to adjust the interface type to transmission type by considering the vehicular environment, thus maximizing the overall network performance. Vehicular environment information could also be utilized to optimize MPTCP scheduling;
- Collaboration of vehicular network with UAVs: Due to the constant movement of vehicles and limited communication links, the connection time between vehicles varies and sometimes can be very short. The introduction of UAVs to vehicular network systems will be able to alleviate this problem to some extent. Since, connection time of connected vehicles could be extended by deploying UAVs appropriately;
- MPTCP with SDN in vehicular networks: Due to the characteristic of centralized control of SDN technology, it has been utilized in vehicular networks to improve network performance. For example, Ran Duo et al. in [98] conducted a study on network handover based on SDN technology in a vehicular network. Likewise, it can also be expected to improve the network performance by introducing SDN technology in a MPTCP vehicular network;
- Security issues: Since multipath TCP has multiple paths to transmit data, therefore the possibility to be attacked by malicious attackers also increase compared to conventional TCP. Security issues are discussed in [30]. They mainly put their efforts on the security issues of connection initiation stage. More security issues should be studied in future work.

## 8. Conclusions

MPTCP has attracted tremendous attention from both academia and industries. A discussion on the use of MPTCP in vehicular environments has become an urgent issue. In this paper, we discussed the existing studies, technical challenges, and open problems regarding MPTCP for vehicular networks. We first conducted a survey on MPTCP and then compared MPTCP with SPTCP. Then the related studies on MPTCP were discussed. After that, we reviewed the existing studies on the use of MPTCP for vehicular networks with detailed discussions on the benefits that MPTCP could bring. We also give a brief review on multipath routing technology for vehicular networks. Finally, we pointed out future research directions on MPTCP for vehicular networks.

## Figures and Tables

**Table 1:** Comparison of single path TCP studies.

	Implementation	Type	Efficiency	RTT Fairness	TCP Fairness	Dynamically Adjusting
Standard TCP	Implementing conservative AIMD and congestion control	Loss-based	Low	Yes	Yes	No
CUBIC	Modifying the increase/decrease parameters of standard TCP more aggressively	Loss-based	High	No	Yes	No
STCP	Modifying the increase/decrease parameters of standard TCP more aggressively	Loss-based	High	No	No	No
BIC-TCP	Modifying the increase/decrease parameters of standard TCP more aggressively	Loss-based	High	No	No	No
Fast TCP	Adjusting the sending rate based on the RTT variation	Delay-based	High	Yes	No	No
BBR	Adjusting the sending rate based on the RTT variation and bandwidth	Congestion -based	High	No	Yes	Yes
CTCP	Adding a delay-based component to standard TCP	Hybrid	High	Yes	Yes	No
QTCP	Sender learn the optimal congestion control according to the network condition	Learning-based	High	Yes	Yes	Yes
TCP-RL	Configuring IW and CC dynamically	Learning-based	High	Yes	Yes	Yes

**Table 2:** Studies on MPTCP congestion control.

Main Metrics or Key Idea	Publication	Research Summary	Limitation/Advantages
Packet loss rate and RTT	Wischik et al. [45]	A mechanism that takes into account the packet loss and RTT in the congestion window adjustment.	TCP fairness/Vulnerable in heterogeneous network
Packet queuing delay	Cao et al. [49]	An approach that formulates the multipath congestion problem and considers packet queuing delay as signal for congestion control.	Sensitive to network change/Weakness in taking available network bandwidth
RTT, retransmission timeout value, packet loss	Honda et al. [52]	A TCP-friendly approach for multipath transport protocol that considers the bottleneck of network.	TCP fairness/Inefficient utilization of network bandwidth
Loss and delay signal	Hassayoun et al. [53]	A proposal that detects shared bottlenecks by loss and delay signals, and makes decision of coupling/decoupling subflows.	Adaptive to network change/No real system evaluation
Delay and loss signal	Singh et al. [54]	A comparison study of different congestion control variants in three different scenarios.	A comparison study
Rate distribution vector and RTT	Li et al. [55]	A proposal that considers the delay difference of different paths in the congestion control.	Minimize the delay difference of paths/Possibility of sacrifice throughput
Goodput	Zhou et al. [56]	An algorithm that consider in-order packets that sent to the application layer as the main metric.	Improving goodput/Possibility of sacrificing slow path throughput
Experience driven	Xu et al. [57]	A DRL-based congestion control framework that consists of LSTM-based representation network, a critic and an actor network.	Dynamically perform congestion control/Need time to train
Adaptive	Naeem et al. [58]	A model free adaptive congestion control framework based on a fuzzy normalized neural network.	Adaptively adjusting congestion window/Take time to train
Experience driven	Li et al. [59]	A proposal that utilizes the congestion rules learnt from observing the environments to adjust the congestion window size of subflows.	Adaptively adjusting window size/TCP unfriendliness
Learning-based	Mai et al. [60]	A proposal that employs deep deterministic policy gradient for learning the optimal congestion control strategies by interacting with the underlying network environment in satellite communications.	Optimizing congestion control strategy/No real system evaluation
Adaptive	Liu et al. [61]	A protocol for NDN, which first predicts the congestion level, and then adjusts the sending rate based on congestion level.	Adaptively adjusting congestion control/Not compatible with IP-based network

**Table 3:** Studies on the MPTCP scheduler.

Main Metrics or Key Idea	Publication	Research Summary	Limitation/Advantages
Sorting every path	C. Casetti et al. [33]	A proposal that sorts each path and transmits the packets in descending order of ranks, so as to provide in-order delivery.	Load balancing/Consider bandwidth only
Delay differences	Raiciu et al. [65]	A proposal that considers the delay differences on different paths by re-injecting the segment causing HoL blocking into the other available subflows.	Considering the delay difference of paths/Reduction of congestion window size
Queuing delays and packet drops	Frommgen et al. [66]	ReMP duplicates packets over all subflows in order to obtain reliability by considering both queuing delays and packet drops.	Reliable transmission/High overhead
Bandwidth	Sebastien Barre [67]	A proposal that selects packets from shared sending buffer and schedules packets on each subflow based on its estimated bandwidth.	Load balancing/Consider bandwidth only
Path capacity	Yang et al. [68]	A proposal that decides scheduling policy based on the estimated path capacity of the subflows.	Load balancing/More extensive experiments are needed
Transmission data	Ferlin et al. [70]	An algorithm that suppresses RTTs by limiting the amount of transmission data.	Suppressing RTTs/Reduction of transmission data
Data-sorting cost and transfer time	Hasegawa et al. [71]	A data distribution method for reducing the data-sorting cost of the receiver buffer and a path-failure detection and recovery mechanism for preventing data transfer stalling.	Reducing data sorting cost and improving throughput/Without considering loss rate when distributing data
Download time	Guo et al. [72]	DEMS, a scheduler aims at reducing the data chunk download time over multiple paths, especially benefiting medium size files and small size web pages.	Reducing download time/Only focusing on two paths
HoL	Ferlin et al. [73]	A proposal that aims to minimize head-of-line (HoL) blocking at the receiver side in heterogeneous networks.	Minimizing HoL/More elements of heterogeneity should be considered
RTT	Mirani et al. [74]	A proposal that not only estimates the arrival time of packets on transmitting path, but also estimates the data delivery time on the other paths, in order to achieve the synchronization of data reception through all paths.	Estimating the arrival time of packets on all paths/Consider RTT only
RTT and loss rate	Xue et al. [75]	An algorithm that fixes the scheduling value based on the estimated data amount on all transmitting paths.	Reducing delay difference of paths/Vulnerability in highly dynamic networks
RTT, loss rate, and cwnd	Ni et al. [76]	An algorithm that calculates the latency based on the scheduling path and the states of other transmitting paths.	Reducing delay difference of paths/Vulnerability in bursty losses
Delay	Hwang et al. [77]	A proposal that freezes a slow path temporarily in order to ensure a fast transmission of data for small flows when the latency difference of the slow path and fast path is significant.	Improving overall transmission rate/Underutilization of available paths
Data distribution way	Paasch et al. [78]	A generic framework that can change the data distribution way over the subflows.	Capability of testing different schedulers of MPTCP/Limited set of traffic classes
Delay	Chayyasith et al. [79]	A proposal that improves the link utilization rate and eliminates the out-of-ordered packets in the receiver buffer.	Improving link utilization rate and in-order packets arrival rate/TCP fairness has not been tested yet
Learning-based	Zhang et al. [80]	A scheduling policy that applies reinforcement learning to enable adaptive scheduling for various network conditions and traffic patterns.	Dynamically generate packet scheduling policy/Need time to train
Learning-based	Luo et al. [81]	A framework to enhance MPTCP scheduling performance in the asymmetric path by applying the best policy to choose the best path to transmit data.	Choosing the best transmission path/More factors should be considered such as RTT
Learning-based	Rosello [82]	A proposal that utilizes deep reinforcement learning agent to interacts with the environments and learn the strategy for distributing the packet optimally.	Learning the strategy for distributing packet optimally/Improvement is not significant
Congestion balancing	Khalili et al. [83]	A study on MPTCP performance issues addressing the congestion balancing across different paths without prior knowledge.	Congestion balancing without prior knowledge/Minimum probing traffic rate should be improved

**Table 4:** Studies on MPTCP in a vehicular ad hoc network.

Research Focus	Publication	Research Summary
V2I scenario	Nigel et al. [100]	A discussion on MPTCP for V2I scenarios.
Transmission stability	Zhu et al. [101]	A study for MPTCP transmission stability in vehicular networks.
Throughput	Cloud et al. [102]	A network coding based MPTCP for mobile device communications in heterogeneous networks.
	Bai et al. [103]	A network coding based MPTCP to solve communication bottleneck in VANETs.
	Zhao et al. [104]	A proposal that apply Q-Learning on MPTCP to schedule the path and reduce the buffer blocking.
	Nan et al. [105]	A congestion control algorithm that considers the message priority and throughput.
High speed train scenario	Dong et al. [106]	A discussion of MPTCP for high speed train scenarios.
Architectural design	Lee et al. [107]	A collaborative MPTCP architecture that considers the device status and user preference.
	Rene et al. [108]	A mechanism that combines MPTCP with DPI to implement selective discarding on the sender side and partial reliability on the receiver side.

## Data Availability

Not applicable.

## Abbreviations

The following abbreviations are used in this manuscript:

ACCP	Adaptive Congestion Control Protocol
AI	Artificial Intelligence
AIMD	Additive Increase/Multiplicative Decrease
AODV	Ad Hoc On-Demand Distance Vector
API	Application Programming Interface
BBR	Bottleneck Bandwidth and Round Trip Propagation Time Congestion Control
BIC-TCP	Binary Increase Congestion Control-Transmission
CAR	Connectivity Aware Routing
CC	Congestion Control
CDN	Content Delivery Network
CLMR	Cross Layer Multipath Routing
CMT-SCTP	Concurrent Multipath Transfer-Stream Control Transmission Protocol
CTCP	Compound Transmission Control Protocol
Cwnd	Congestion Window
DCCP	Data Congestion Control Protocol
DHFT	Dual Homed FatTree
DoS	Denial of Service
DPI	Deep Packet Inspection
DQN	Deep Q Network
DRL	Deep Reinforcement Learning
DSDV	Dynamic Destination Sequenced Distance Vector
DSR	Dynamic Source Routing
DTN	Delay Tolerant Network
DWC	Dynamic Window Coupling
ECMP	Equal Cost Multipath Protocol
EWTCP	Equal Weighted Transmission Control Protocol
GCS	Ground Control System
GPS	Global Positioning System
HMAC	Hash Based Message Authentication Code
HoL	Head of Line
HTTP	Hypertext Transfer Protocol
IEEE	Institute of Electrical and Electronic Engineers
IETF	Internet Engineering Task Force
IoT	Internet of Things
IP	Internet Protocol
IW	Initial Window
LIA	Linked Increases Algorithm
LISP	Location/Identifier Separation Protocol
LSTM	Long Short Term Memory
LTE	Long Term Evolution
MAC	Media Access Control
MBMPR	Map Based Multipath Routing
MCs	Multiplexed Connections
mmWave	Millimeter Wave
MPTCP	Multipath Transmission Control Protocol
ND	Network Coding
NDN	Named Data Networking
OLIA	Opportunistic Linked Increases Algorithm
PHY	Physical Layer
PSO	Practice Swarm Optimization
QoS	Quality of Service
QTCP	Q-learning framework with TCP
QUIC	Quick UDP Internet Connection Protocol
RAID	Redundant Array Inexpensive Disks
RL	Reinforcement Learning
RSU	Road Side Unit
RR	Round Robin
RTT	Round Trip Time
SCTP	Stream Control Transmission Protocol
SDN	Software Defined Network
SPTCP	Single Path Transmission Control Protocol
SRTT	Shortest Smoothed RTT
STCP	Scalable Transmission Control Protocol
TCP	Transmission Control Protocol
TORA	Temporally Ordered Routing Algorithm
UAV	Unmanned Aerial Vehicle
UDP	User Datagram Protocol
VANETs	Vehicular Ad Hoc Networks
VoIP	Voice over Internet Protocol
V2I	Vehicle to infrastructure
wVegas	Weighted Vegas
